# Diversity of Nematoda and Digenea from different species of characiform fishes from Tocantins River, Maranhão, Brazil

**DOI:** 10.1590/S1984-29612022038

**Published:** 2022-07-22

**Authors:** Melissa Querido Cárdenas, Márcia Cristina Nascimento Justo, Amanda da Rocha Paula Reyes, Simone Chinicz Cohen

**Affiliations:** 1 Laboratório de Helmintos Parasitos de Peixes, Instituto Oswaldo Cruz, Fundação Oswaldo Cruz – Fiocruz, Rio de Janeiro, RJ, Brasil

**Keywords:** Nematoda, Digenea, Characiformes, Tocantins River, Maranhão, Brazil, Nematoda, Digenea, Characiformes, rio Tocantins, Maranhão, Brasil

## Abstract

During a survey of the helminth fauna of characiform fishes from the Tocantins River, Brazil, 185 fish specimens from 22 species were studied. Twelve species of Nematoda and nine species of Digenea were collected. Some of these helminth species were reported for the first time in their hosts, thus representing new host records: *Procamallanus* (*Spirocamallanus*) sp. in *Bivibranchia notata*, *Brycon pesu*, *Chalceus macrolepidotus*, *Hemiodus microlepis* and *Hemiodus unimaculatus*; *Procamallanus* (*Spirocamallanus*) *inopinatus* in *Triportheus elongatus*; *Goezia* sp. (larva) in *Boulengerella cuvieri*; *Rhabdochona acuminata* in *Brycon pesu* and *Triportheus trifurcatus*; *Raphidascaris* sp. (larva) in *Caenotropus labyrinthicus*; *Cosmoxynema vianai* in *Cyphocharax gouldingi*; *Rondonia rondoni* in *Leporinus fasciatus* and *Mylesinus paucisquamatus*; *Klossinemella iheringi* in *Mylesinus paucisquamatus*; Cucullanidae gen. sp. in *Myloplus rubripinnis*; *Rhabdochona* sp. in *Triportheus elongatus*; *Alphamphistoma* sp. in *Myleus setiger*; *Chalcinotrema* sp. in *Cyphocharax gouldingi*; *Pacudistoma guianense* in *Hemiodus unimaculatus* and *Myleus torquatus*; *Pseudocladorchis cylindricus* in *Hemiodus unimaculatus*; *Dadaytrema oxycephala* in *Mylesinus paucisquamatus*; *Travassosinia dilatata* in *Myloplus asterias*; and *Genarchella genarchella* in *Raphiodon vulpinus*. Studies identifying new hosts and new localities for parasites have contributed to the knowledge of local biodiversity. A list of previous records of helminths included in the present study, providing hosts, localities, and references, is presented here.

## Resumo

Durante a pesquisa da fauna helmintológica de Characiformes do rio Tocantins, Maranhão, Brasil, 185 peixes pertencentes a 22 espécies foram estudados. Doze espécies de Nematoda e nove espécies de Digenea foram coletadas. Algumas espécies de helmintos estão sendo registradas pela primeira vez em seus hospedeiros, representando novos registros: *Procamallanus* (*Spirocamallanus*) sp. em *Bivibranchia notata*, *Brycon pesu*, *Chalceus macrolepidotus*, *Hemiodus microlepis* e *Hemiodus unimaculatus*; *Procamallanus* (*Spirocamallanus*) *inopinatus* em *Triportheus elongatus*; *Goezia* sp. (larva) em *Boulengerella cuvieri*; *Rhabdochona acuminata* em *Brycon pesu* e *Tripotheus trifurcatus*; *Raphidascaris* sp. (larva) em *Caenotropus labyrinthicus*; *Cosmoxynema vianai* em *Cyphocharax gouldingi*; *Rondonia rondoni* em *Leporinus fasciatus* e *Mylesinus paucisquamatus*; *Klossinemella iheringi* em *Mylesinus paucisquamatus*; Cucullanidae gen. sp. em *Myloplus rubripinnis*; *Rhabdochona* sp. em *Triportheus elongatus*; *Alphamphistoma* sp. em *Myleus setiger*; *Chalcinotrema* sp. em *Cyphocarax gouldingi*; *Pacudistoma guianense* em *Hemiodus unimaculatus* e *Myleus torquatus*; *Pseudocladorchis cylindricus* em *Hemiodus unimaculatus*; *Dadaytrema oxycephala* em *Mylesinus paucisquamatus*; *Travassosinia dilatata* em *Myloplus asterias*; e *Genarchella genarchella* em *Raphiodon vulpinus*. Estudos que identificam novos hospedeiros e novas localidades para parasitos têm contribuído para o conhecimento da biodiversidade local. Uma lista dos registros prévios dos helmintos incluídos no presente estudo, com hospedeiros, localidades e referências é apresentada.

## Introduction

South America presents a high ichthyological diversity, which shows the huge dimensions of the challenge involved in acquiring basic knowledge of the helminth fauna of fish in this continent (Luque et al., 2017). Despite recent increases in biodiversity research, parasites are still a neglected group, even though they represent a key role in the functioning of ecosystems (Marcogliese & Cone, 1997; Adlard et al., 2015; Rocha et al., 2016; Acosta et al., 2020). Thus, there is a risk that a large number of parasite species may be lost even before their existence becomes known or before their risk of extinction is understood. This emphasizes the importance of taxonomy and species cataloging (Moravec, 2007; Rocha et al., 2016).

The Tocantins-Araguaia basin is one of the most important hydrographic regions of Brazil. It is an area of endemism for several neotropical freshwater fish groups, as siluriforms and characiforms, as identified by several authors (e.g. Vari, 1988; Menezes & Lucena, 1998; Lima & Moreira, 2003), and an area of high diversity (Santos et al., 2004; Lucinda et al., 2007; Abell et al., 2008). The fish species present in this basin are a mixture of species from the Amazon River basin, either from its plain or from tributaries of the lower part of the river, and species from the central Brazil and Guiana shields (Ferreira et al., 2011). The Tocantins River arises in the state of Goiás, in central Brazil, and runs northwards through different sedimentary basins for 2,500 km across the states of Tocantins, Maranhão, and Pará. It is formed by the confluence of two main tributaries: the Paranã and Maranhão Rivers (Provete, 2013).

Neotropical freshwater fish constitute the most diverse continental vertebrate fauna on Earth, with more than 6,200 named species, assigned by taxonomists to 854 genera, 95 families, and 39 orders (Albert et al., 2020). The order Characiformes is the largest group of freshwater fish, with at least 2,300 valid species distributed in 520 genera. Characiforms are one of the largest components of the freshwater fish fauna worldwide and are distributed across the New World and Africa, but mainly in the Neotropical Region, and specifically from the south of North America to South America (Froese & Pauly, 2022). They inhabit a range of ecosystems extending from the swiftly flowing rivers and streams of the Andean piedmont and cordilleras of the Neotropics through to the lentic backwaters of lowland floodplains in the Americas and Africa. Within these habitats, characiforms range from dozens of miniature and diminutive species through to hundreds of midsized and to giant species. Neotropical characiforms form a monophyletic clade and are grouped in 14 families (Oliveira et al., 2011).

Despite the rich diversity of fish species, the parasite fauna of fish from the Tocantins River remains poorly known, although some research on new species of helminths and new geographical records of known species has been published (Moravec & Thatcher, 1999; Fernandes et al., 2013; Lacerda et al., 2013; Cárdenas et al., 2019; Cohen et al., 2020). Thus, the aim of the present study was to report on the fauna of Nematoda and Digenea parasitizing characiform fishes in the Tocantins River, Maranhão, Brazil.

## Material and Methods

**Figure 1:**
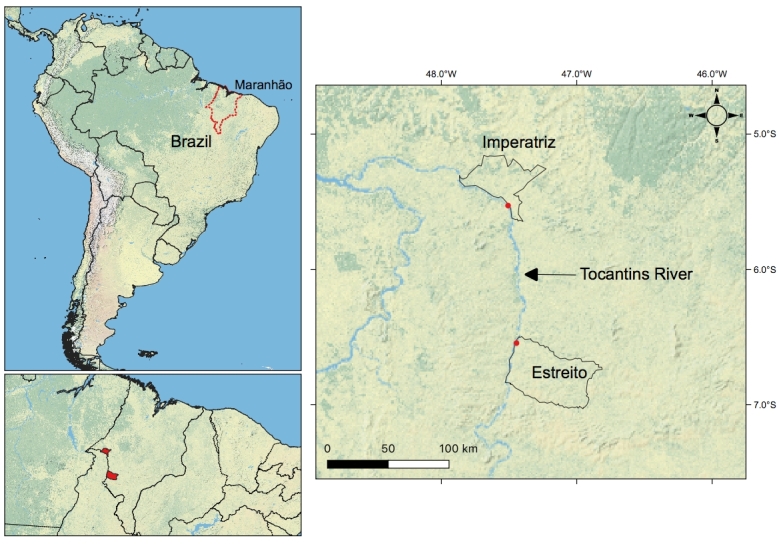
Map of Middle Tocantins River showing the collection points, Maranhão, Brazil, in the municipalities of Estreito and Imperatriz, Maranhão.

During August 2010, a survey of the helminth fauna in fish in the Tocantins River, state of Maranhão, municipalities of Estreito, Brazil (06º 33' 38” S, 47º 27' 04” W) and Imperatriz (05º 31' 35” S, 47º 29' 30” W) was carried out (Figure 1). From this, 185 characiform specimens belonging to 22 species were studied. These were acquired with the aid of local fishermen, and identifyed by Dr. Gustavo Wilson Nunam (*in memoriam*) from the “Museu Nacional, Departamento de Vertebrados, Ictiologia, UFRJ” and were examined for parasites immediately upon capture. Internal organs were separated in Petri dishes containing 0.65% NaCl and were examined with the aid of a stereoscopic microscope. The nematodes and digeneans found were washed in 0.65% NaCl and fixed in AFA (2% glacial acetic acid, 3% formaldehyde and 95% ethanol 70° GL). Nematodes were cleared with lactophenol or glycerin for examination under an optical microscope (Amato & Amato, 2010). The digeneans were stained with Langeron’s alcoholic acid carmine, dehydrated in an ethyl alcohol series, cleared in clave oil and mounted in Canada balsam as permanent slides (Eiras et al., 2006). The helminths were observed using a Zeiss Axioscope 2 microscope equipped with a camera lucida.

A list of previous records of helminths with valid names included in the present study, reported from South America, is presented based on information from published articles obtained from databases. Parasites are organized according to class, order and family, within which species are presented in alphabetical order, including geographical distribution and references. Fish species are arranged in alphabetical order and valid names are taken from FishBase (Froese & Pauly, 2022).

## Results and Discussion

In the present study, twelve species of Nematoda and nine species of Digenea were collected. Some nematodes species were identified at genus level due to they are consisted of immature specimens or only females, and the digeneans *Alphamphistoma* sp. and *Chalcinotrema* sp. were found in low numbers, unabling the specific identification. All hosts were parasitized by at least one species of digenean and/or one species of nematode. The prevalences and intensities were low, and this can be explained by the low number of specimens of hosts examined. However, two species, *Rondonia rondoni* Travassos, 1920 and *Klossinemella iheringi* (Travassos, Artigas & Pereira, 1928) parasitizing *Mylesinus paucisquamatus* Jégu & Santos and the latter also *Leporinus* sp. presented the highest intensity of infection (911 and 1,986, respectively) (Table 1).

**Table 1:** Different host species with standard body length and weight in parenthesis, parasites, number of parasitized hosts/ number of examined hosts (NP/NE), range of infection (R), total number of parasites (TNP), site of infection (SI) [(I) intestine, swim bladder (SW), stomach (S), gall bladder (GB)] and development stage (DS) [adult (A), larva (L), metacercaria (M)], of nematodes and digeneans collected from characiform fishes from Tocantins River, Maranhão, Brazil.

**Host**	**Parasite**	**NP/NE**	**R**	**TNP**	**SI**	**DS**
*Bivibranchia notate* Vari & Goulding, 1985 (91-107 g; 12.5-18.9 cm)	**Nematoda** *Procamallanus* (*Spirocamallanus*) sp.*	1/ 17	0-1	1	I	A
*Boulengerella cuvieri* (Spix & Agassiz, 1829) (179-408 g; 37.4-512.2cm)	**Nematoda** *Goezia* sp.*	1/ 13	0-3	3	I	L
*Brycon pesu* Müller & Troschel, 1845 (85-112 g; 14.2-34.6 cm)	**Nematoda** *Rhabdochona acuminata* (Molin, 1860)*	1/ 10	0-2	2	I	A
	*Procamallanus* (*Spirocamallanus*) sp.*	1/ 10	0-1	1	I	A
*Bryconops alburnoides* Kner, 1858 (101-107 g; 16.9-19.6 cm)	**Nematoda** *Procamallanus* sp.	1/ 6	0-1	1	I	A
*Caenotropus labyrinthicus* (Kner, 1858) (111-141 g; 36.2-71.3 cm)	**Nematoda** *Raphidascaris* sp.*	1/ 8	0-5	5	I	L
*Chalceus macrolepidotus* Cuvier, 1818 (95-128 g; 23.8-51.3 cm)	**Nematoda** *Procamallanus* (*Spirocamallanus*) sp.*	1/ 7	0-1	1	I	A
*Cyphocharax gouldingi* Vari, 1992 (132-149 g; 69-89 cm)	**Digenea** *Chalcinotrema* sp.* Austrodiplostomum compactum*	1/ 8 1/ 8	0-3 0-4	3 4	I GB	A M
	**Nematoda** *Cosmoxynema vianai* Travassos, 1949*	1/ 8	0-1	1	I	A
*Hemiodus microlepis* Kner, 1858 (99-163 g; 29-99.3 cm)	**Nematoda** *Procamallanus* (*Spirocamallanus*) *inopinatus*Travassos, Artigas & Pereira, 1928	1/ 9	0-3	3	I	A
	*Procamallanus* (*Spirocamallanus*) sp.	1/ 9	0-4	4	I	A
*Hemiodus unimaculatus* (Bloch, 1794) (102-179 g; 18.9-154.2 cm)	**Digenea** *Pacudistoma guianensis* Thatcher & Jégu, 1998*	1/ 22	0-6	6	I	A
	*Pseudocladorchis cylindricus* (Diesing, 1836)*	1/ 22	0-1	1	I	A
	**Nematoda** *Procamallanus* (*Spirocamallanus*) sp.*	1/ 22	0-1	1	I	A
*Hoplias malabaricus* (Bloch, 1794) (204-208 g; 167.2-251.2 cm)	**Nematoda** *Cystidicoloides* sp.	1/ 3	0-1	1	S	A
*Leporinus fasciatus* (Bloch, 1794) (113-130 g; 23-36.6 cm)	**Nematoda** *Rondonia rondoni* Travassos, 1920*	1/ 2	0-2	2	I	A
*Leporinus friderici* (Bloch, 1794) (204-208 g; 167.2-251.2 cm)	**Digenea** *Genarchella genarchella* Travassos, 1928	1/ 3	0-1	1	I	A
*Leporinus* sp. (51-162 g; 44.6-93.6 cm)	**Digenea** *Prosthenhystera obesa* (Diesing, 1850)	1/ 9	0-1	1	BV	A
	**Nematoda** *Rondonia rondoni*	1/ 8	0-1986	1986	I	A
*Mylesinus paucisquamatus* Jégu & Santos, 1988 (123-155 g; 65.3-158.2 cm)	**Nematoda** *Klossinemella iheringi* (Travassos, Artigas & Pereira, 1928)*	2/ 7	435-735	1220	I	A
	**Nematoda** Rondonia rondoni*	2/ 7	785-911	1696	I	A
	**Digenea** *Dadaytrema oxycephala* (Diesing, 1836)*	1/ 7	0-1	3	I	A
*Myloplus asterias* (Müller & Troschel, 1844) (136 g; 131.7cm)	**Digenea** *Travassosinia dilatata* (Daday, 1907)*	1/ 1	1	9	I	A
*Myleus setiger* Müller & Troschel, 1844 (150 g; 151.7 cm)	**Digenea** *Alphamphistoma* sp.	1/ 1	1	1	I	A
*Myloplus torquatus* (Kner, 1858) (25.9-152 g; 90.6-184.2 cm)	**Digenea** *Dadaytrema oxycephala*	1/ 3	0-1	3	I	A
	*Pacudistoma guianensis* Thatcher & Jégu, 1998*	1/ 3	0-1	1	I	A
*Myloplus rubripinnis* (Müller & Troschel, 1844) (77 g; 26.6 cm)	**Nematoda** Cucullanidae gen. sp.*	1/ 1	1	1	I	A
*Triportheus elongatus* (Günther, 1864) (77-166 g; 10.9-90.7 cm)	**Nematoda** *P* (*S*.) *inopinatus*	2/ 25	1-7	8	I	A
	*Rhabdochona* sp.*	1/ 25	0-1	1	I	A
*Triportheus trifurcatus* Castelnau, 1855 (102-259 g; 34-115.8 cm)	**Nematoda** Rhabdochona acuminata*	1/ 23	0-20	20	I	A
*Raphiodon vulpinus* Spix & Agassiz, 1829 (210-359 g; 133-264 cm)	**Digenea** *Genarchella genarchella* ^*^	1/ 5	0-1	1	S	A
*Serrasalmus maculatus* Kner, 1858 (85-153 g; 15.6-121.3 cm)	**Nematoda** *Procamallanus* (*S*.) *inopinatus*	2/ 7	1-2	3	I	A

* New host record

*Rondonia rondoni* and *K. iheringi* belong to the family Atractidae and usually occur in fish hosts in large numbers (Dias et al., 2004; Campos et al., 2009). Although the life cycles of these nematode species are not known, it is highly likely that they are homoxenous, i.e. no intermediate hosts are involved (Moravec, 1998), which would explain the high indices found in the present study considering that they can reach the host easily.

*Procamallanus* Baylis, 1923 is one of the most common nematode genera living on fish in the Neotropical region. To date, *Procamallanus* (*Spirocamallanus*) *inopinatus* Travassos, Artigas & Pereira, 1928 and *Procamallanus* (*Spirocamallanus*) sp. were previously reported in several fish species in South America (see list below). *Procamallanus* (*S*.) *inopinatus* was first described by Travassos et al. (1928) from two undetermined *Leporinus* species found in the Mogi Guaçu River (state of São Paulo, Brazil). This is a widely distributed nematode species in South American freshwater fishes, which mainly parasitizes several characoid fish but also occurs in some other groups of fish that probably serve as postcyclic or paradefinitive hosts (Moravec et al., 1993a; Moravec, 1998). In the present study, *P*. (*S*.) *inopinatus* was found in hosts that had already been recorded, while *Procamallanus* (*S*.) sp. is reported here for the first time in *Bivibranchia notata* Vari & Goulding, *Brycon pesu* Müeller & Troschel, *Hemiodus unimaculatus* (Bloch) and *Chalceus macrolepidotus* Cuvier, which thus represent four new hosts records for this nematode. Until now, the helminth fauna of *C*. *macrolepidotus* was unknown, and *Procamallanus* (*S*.) sp. represents the first helminth record in this fish host.

*Rhabdochona acuminata* (Molin, 1860) Drasche, 1884 was originally described as *Spiroptera acuminata* by Molin (1860), found in *Brycon falcatus* Müeller & Troschel. Travassos et al. (1928) detailed the description and reproduced the figures of Drasche (1884) (who found this nematode in *Barbus* sp. and *B*. *falcatus*); and reported findings of *R. acuminata* (= *Rhabdochona elegans*) in *Tetragonopterus* sp. Subsequently, this species has been reported in several hosts and localities (see list below).

The presence of the nematodes *Cosmoxynema vianai* Travassos, 1949, *Cystidicoloides* sp., *Goezia* sp., *K*. *iheringi*, *R*. *acuminata*, *Rhabdochona* sp., *Raphidascaris* sp. and *R*. *rondoni* represents new geographical records (Tocantins River). This emphasizes the role of fish as definitive hosts for diverse nematode species.

*Dadaytrema oxycephala* (Diesing, 1850), *Prosthenhystera obesa* (Diesing, 1850) and *Genarchella genarchella* Travassos, 1928 are generalist parasites and have been reported in different freshwater fishes (see Kohn et al., 2007). *Dadaytrema oxycephala* is a parasite with wide geographical distribution in South America, where there are 22 records of this digenean parasitizing characiforms and 11 records of it parasitizing siluriform fishes (Negreiros et al., 2020).

*Prosthenhystera obesa* is also widely distributed in characiform and siluriform fish in Brazil and in a single species of characiform fish in Argentina (Kohn et al., 2007; Martins et al., 2012; Vasconcelos et al., 2013; Sabas & Brasil-Sato 2014; Fernandes et al., 2017). Regarding other studies with characiform and siluriform fishes, the low intensity indices for *P. obesa* (only one specimen per fish) observed in the present study were similar to those reported by Martins et al. (2012) in *Leporinus reinhardti* Lütken and *Pimelodus pohli* Ribeiro & Lucena; by Vasconcelos et al. (2013) in *Astyanax* aff. *bimaculatus*; by Brasil-Sato & Pavanelli (2004) in *Pimelodus maculatus* Lacepède; by Sabas & Brasil-Sato (2014) in *P. pohli*; by Brasil-Sato (2002) and Karling et al. (2013) in *Salminus brasiliensis* (Cuvier); and by Fernandes et al. (2017) in *Acestrorhynchus falcirostris* (Cuvier). In those reports, from one to no more than three specimens per fish were found. Considering the relative size of *P. obesa* and the site of infection, higher intensity could be harmful to both the host and the parasite. According to Martins et al. (2012), placement of the parasite in the gall bladder is a factor that controls the intensity. These factors may explain the low intensity found for this helminth in the present study.

Adult forms of *G. genarchella* have been reported in a wide diversity of fish species in Brazil and Argentina (Kohn et al., 2007). In the present study, this parasite presented low intensity (only one specimen per fish), parasitizing *Leporinus friderici* (Bloch) and *Rhaphiodon vulpinus* Spix & Agassiz. Similar results were obtained in other studies in Brazil, e.g., the reports in *Cichla piquiti* Kullander & Ferreira by Franceschini et al. (2013b), in *Hemibrycon surinamensis* Géry by Hoshino et al. (2014), in *A*. *falcirostris* by Fernandes et al. (2017) and in *Megaleporinus obtusidens* (Valenciennes) by Wendt et al. (2018). However, the mean intensities of *G. genarchella* in the stomach and intestine of *Peckoltia braueri* (Eigenmann) presented by Cardoso et al. (2017) were slightly higher (6.0 and 24, respectively) than those of the present study. The mean abundance values for this parasite reported by Hoshino & Tavares-Dias (2019) in *H. surinamensis* were 25.3 in the rainy season and 6.6 in the dry season.

*Genarchella genarchella* has mollusks and Cypriniformes species as intermediate hosts, and Characiformes and Siluriformes species as definitive hosts (Lefebvre & Poulin, 2005). The finding of adult forms of *G. genarchella* in the present study confirms that the characiforms *L. friderici* and *R. vulpinus* act as the definitive hosts for this digenean.

*Pacudistoma guianense* was originally described by Thatcher and Jégu in 1998, from the intestine of *Myleus ternetzi* (Norman) in French Guyana. The present report provides the first record of this species in Brazil, and with a new host record, *Hemiodus unimaculatus* (Bloch). The digeneans *Alphamphistoma* sp., *Chalcinotrema* sp., *Dadaytrema oxycephala*, *P. obesa* (Diesing, 1850), *Pseudocladorchis cylindricus* (Diesing, 1836) and *Travassosinia dilatata* (Daday, 1907) are reported here for the first time in the Tocantins River. The records of these species in different localities and from characiform hosts are presented here in a list, and these records demonstrate the wide geographical distribution of these species in South America. Trematodes form an important part of the biological diversity of different ecosystems and represents the second-richest group of helminths in fish species in South America (Luque et al., 2017). In freshwater fishes, this group is the most frequent, followed by nematodes, in terms of the number of parasite associations recorded in Neotropical fishes (Luque & Poulin, 2007).

Knowledge of the parasitic fauna of wild fish constitutes a biodiversity assessment tool. It enables better understanding of host biology and the relationship between hosts and their parasites, which may form environmental indicators (Takemoto et al., 2009; Tavares-Dias et al., 2013; Shah et al., 2014). Studies identifying new hosts and new localities for parasites have contributed to knowledge of local biodiversity, as well as to understanding the evolution of parasites and their hosts (Lacerda et al., 2008).

Some of the nematode and digenean parasites of Characiformes are reported here for the first time in their hosts, thus representing new hosts and geographical records (Table 1), demonstrating a diversity of parasitic species in different host species in the Tocantins-Araguaia basin. These data corroborate the prediction that many helminth species are still to be described and/or reported, and contribute to significant knowledge of South American biodiversity. The previous reports of these nematodes and digeneans in characiform hosts that were studied here are presented in the list below, including data from the present study.

List of previous records of helminths included in the present study, providing hosts, localities and references. Abbreviation of the Brazilian states: AC: Acre, AL: Alagoas, AP: Amapá, AM: Amazonas, BA: Bahia, ES: Espírito Santo, GO: Goiás, MA: Maranhão, MT: Mato Grosso, MS: Mato Grosso do Sul, MG: Minas Gerais, PA: Pará, PB: Paraíba, PR: Paraná, PE: Pernambuco, RJ: Rio de Janeiro, RN: Rio Grande do Norte, RS: Rio Grande do Sul, RO: Rondonia, SC: Santa Catarina, SP: São Paulo, SE: Sergipe.

### DIGENEA

#### *Alphamphistoma* sp.

**Host: *Myleus setiger* (present study)**

**Locality: BRAZIL: Tocantins River (MA) (present study).**

*Austrodiplostomum compactum* (Lutz, 1928) Dubois, 1970 (Metacercariae)

**Hosts:***Acestrorhynchus lacustris* (Lütken), ***Cyphocharax gouldingi* Vari (present study),** *Cyphocharax gilbert* (Quoy & Gaimard), *Hoplias* aff. *malabaricus*, *Hoplias malabaricus* (Bloch), *Hoplias* spp., *Leporinus amblyrhynchus* Garavello & Britski, *Metynnis maculatus* (Kner), *Piaractus mesopotamicus* (Holmberg), *Potamorhina latior* (Spix & Agassiz), *Potamorhina pristigaster* (Steindachner), *Prochilodus lineatus* (Valenciennes), *Pygocentrus nattereri* Kner, *Schizodon borellii* (Boulenger), *S. intermedius* Garavello & Britski, *S. nasutus* Kner, *Serrasalmus maculatus* Kner.

**Localities: BRAZIL:** Lakes Ananá, Araçá, Baixio, Catalão, Iauara, Maracá, Preto (Solimões River floodplain), lake of Purus River (AM); **Tocantins River (MA) (present study);** Carioca Lake (MG); Paraná River, Porto Rico Region, Rosana reservoir (Paranapanema River), Upper Paraná River floodplain (PR); Dam of the Water Treatment Station (ETA), Guandu River (RJ); Batalha River, Chavantes reservoir (medium Paranapanema River), Jurumirim reservoir (Paranapanema River), Santa Bárbara River (mid Tietê river), Mogi Guaçu River (SP).

**References:**Abdallah et al. (2005), Belei et al. (2013), Corrêa et al. (2020), Gião et al. (2020), Lehun et al. (2020), Machado et al. (2005), Morais et al. (2011), Pavanelli et al. (1997), Pedro et al. (2016), Ramos et al. (2013, 2016), Santos et al. (2012), Vital et al. (2016), Yamada et al. (2008).

#### *Chalcinotrema* sp.

**Host: *Cyphocharax gouldingi* (present study)**

**Locality: Brazil: Tocantins River (MA) (present study).**

*Dadaytrema oxycephalum* (Diesing, 1850) Vaz, 1932

**Hosts:***Brycon orbignyanus* (Valenciennes); *Colossoma macropomum* (Cuvier); ***Mylesinus paucisquamatus* (present study)**; *Myleus micans* (Lütken); *Myleus* sp.; *Myloplus asterias* (=*Myleus asterias*); *M*. *rhomboidalis* (=*Myleus rhomboidalis*) (Cuvier); ***M. torquatus* (*=Myletes torquatus*, *Myleus torquatus*) (Kner) (present study)**; *Mylossoma aureum* (Spix & Agassiz); *M*. *duriventre* (Cuvier); *Piaractus brachypomus* (=*Colossoma bidens*) (Cuvier); *P. mesopotamicus* (=*Colossoma mitrei*); *Pterodoras granulosus* (Valenciennes); *Salminus brasiliensis* (*=Salminus maxillosus*) (Cuvier); *Salmo* sp.

**Localities: ARGENTINA:** Medium of Paraná River, Corrientes; Colastine River (Medium Paraná River) and Paraná-Guazú River (Lower Paraná River). **BRAZIL:** Amazon River, Janauacá Lake (Manaus), Jari River (a tributary of the Amazon River) (AM); Macapá (AP); **Tocantins River, MA (present study);** São Francisco River (MG); Aquidauana River, Cuiabá River, Miranda River, Paraguay River, Paraná River, Pantanal (MS); Amazon River, Cachimbo (PA); Upper Paraná River (PR); Mogi Guaçu River, Paranapanema River (SP); **VENEZUELA:** Caura River.

**References:**Brasil-Sato & Santos (2003), Campos et al. (2009), Conroy (1985, apud Kohn et al., 2007), Fernandes et al. (2019), Hamann (1982a, b), Heyneman et al. (1960), Kohn & Fernandes (1987); Nuñez et al. (2017), Oliveira & Tavares-Dias (2016), Oliveira et al. (2019), Pantoja et al. (2019), Thatcher (1979, 1999), Travassos et al. (1928), Vaz (1932), Vicente et al. (1978).

#### *Genarchella genarchella* Travassos, 1928

**Hosts:***Acestrorhynchus falcirostris*; *Charax stenopterus* (=*Asiphonichthys stenopterus*) (Cope); *Cynopotamus humeralis* (*=Acestrorhamphus* sp.) [*sic*]; *Hemibrycon surinamensis*; ***Leporinus friderici* (Bloch) (present study);** *Megaleporinus obtusidens* (=*Leporinus obtusidens*); *Oligosarcus jenynsii* (Günther); ***Rhaphiodon vulpinus* Spix & Agassiz (present study);** *Salminus brasiliensis* (*=Salminus maxillosus*).

**Localities: ARGENTINA:** Paraná River, Puerto Itália, Corrientes. **BRAZIL:** Emas Waterfall, Pirassununga (SP); Mogi Guaçu River, Emas Experimental Station, Pirassununga, (SP); Floodplain Lake of the Solimões River (Ananá), (AM); Lake Guaíba (RS); Igarapé Fortaleza Basin (AP); **Tocantins River (MA) (present study).**

**References:**Fernandes et al. (2017), Hamann (1986, 1989), Hoshino et al. (2014), Kohn & Fernandes (1988), Kohn et al. (1990), Szidat (1956), Travassos & Kohn (1965), Travassos et al. (1928), Wendt et al. (2018).

#### *Pacudistoma guianense* Thatcher & Jégu, 1998

**Hosts: *Hemiodus unimaculatus* (present study),***Myloplus ternetzi* (=*Myleus ternetzi*) (Norman); ***M. torquatus* (Kner) (present study).**

**Localities: BRAZIL: Tocantins River (MA); FRENCH GUYANA:** Sinnamary River.

**Reference:**Thatcher & Jégu (1998).

#### *Prosthenhystera obesa* (Diesing, 1850) Travassos, 1922

**Hosts:***Acestrorhynchus falcatus* (Bloch); *A*. *falcirostris*; *Astyanax bimaculatus* (Linnaeus); *Boulengerella cuvieri* (=*Xiphostoma cuvieri*) (Spix & Agassiz); *Brycon orthotaenia* (=*Brycon lundii; Triurobrycon lundii*) Günther; *Brycon* sp.; *Charax gibbosus* (Linnaeus); *Cynopotamus amazonum* (=*Cynopotamus amazanus*) (Günther); *Galeocharax humeralis* (*=Acestrorhamphus* sp.) (Valenciennes); *Hypomasticus copelandii* (*=Leporinus copelandii*) (Steindachner); *Leporellus vittatus* (Valenciennes); *Leporinus friderici* (=*Leporinus friderici friderici*); ***Leporinus* sp. (present study)**; *Oligosarcus* sp. (=*Acesthrorhampus* sp.); *Psalidodon fasciatus* (=*Astyanax fasciatus*) (Cuvier); *Salminus brasiliensis* (=*Salminus brevidens, Salminus maxillosus*); *S. franciscanus* (= *Salminus brevidens*) Lima & Britski; *S*. *hilarii* Valenciennes.

**Localities: ARGENTINA:** Delta of Paraná River, Irigoyen Channel, Talavera Island. **BRAZIL:** Cruzeiro do Sul (AC); Lake Ananá (Solimões River) (AM); Rio Grande (BA); Jupranã Lagoon (ES); **Tocantins River (MA) (present study)**; Lassance, Pirapora, São Francisco River (MG); Porto Esperança (MS); Porto Esperança (Paraguai River), Porto São João (Cuiabá River), Salobra (MT); Emas (Pirassununga), Ilha Seca, Mogi Guaçu River, Porto Tibiriçá (SP); Paraná River, Foz do Iguaçu (PR).

**References:**Brasil-Sato (2002), Diesing (1850), Fernandes et al. (2017), Isaac et al. (2000), Karling et al. (2013), Kloss (1966), Kohn & Fernandes, (1981), Kohn et al. (1997), Lunaschi & Sutton (1995), Pavanelli et al. (1992, apud Kohn et al., 2007), Travassos (1941), Travassos & Kohn (1965), Travassos (1922), Travassos et al. (1928), Virgilio et al. (2021).

#### *Pseudocladorchis cylindricus* (Diesing, 1836) Daday, 1907

**Hosts: *Hemiodus unimaculatus* (present study)**; *Mylesinus paraschomburgkii* Jégu, Santos & Ferreira; *Myloplus ternetzi* (=*Myleus ternetzi*); *Mylossoma aureum*; *Piaractus brachypomus* (= *Colossoma bidens*); *Pterodoras granulosus*.

**Localities: BRAZIL:** Uatumã, Pitinga and Capucapu Rivers (AM); Araguari River (AP); **Tocantins River (MA) (present study)**; (MT); Trombetas River (PA); **FRENCH GUYANA:** Sinnamary River.

**References:**Daday (1907), Diesing (1836), Thatcher & Jégu (1996, 1998), Travassos et al. (1928), Viana (1924).

#### *Travassosinia dilatata* (Daday, 1907) Vaz, 1932

**Hosts: *Myloplus asterias* (present study)**; *Myleus micans*; *Myleus* sp.; *Piaractus brachypomus* (=*Colossoma brachypomus*); *P*. *mesopotamicus* (= *Colossoma mitrei*); *Salminus brasiliensis*.

**Localities: ARGENTINA:** Paraná-Guazú River, Entre Ríos Province; Medium Paraná River; **BRAZIL**: São Francisco River (MG); Cachimbo (PA); Paranapanema River (SP); **Tocantins River (MA) (present study).**

**References**: Brasil-Sato & Santos (2003), Daday (1907), Hamann (1982a), Nuñez et al. (2017), Travassos et al. (1928), Vaz (1932), Vicente et al. (1978).

### NEMATODA

#### *Cosmoxynema vianai* Travassos, 1949

**Hosts:***Curimatella meyeri* (Steindachner); *Cyphocharax gilbert*; ***C. gouldingi* (present study);** *C. naegelii* (Steindachner); *Pseudocurimata* sp.; *Steindachanerina brevipinna* (Eigenmann & Eigenmann); *S. elegans* (Steindachner); *S. insculpta* (Fernández-Yépez).

**Localities: BRAZIL:** Cruzeiro do Sul (AC); Barra Seca River, Juparanã Lagoon (ES); **Tocantins River (MA) (present study);** Upper Paraná River (PA); Paraná River (Guaira), Tributaries Guairacá and Corvo (lower Paranapanema river) (PR); Guandu River (RJ); Mogi Guaçu River, Peixe River (SP).

**References:**Abdallah et al. (2005, 2012), Ceschini et al. (2010a, b), Eiras et al. (2010), Luque et al. (2011), Moravec et al. (1992), Takemoto et al. (2009), Travassos (1948), Vicente et al. (1985), Virgilio et al. (2021), Yamaguti (1961).

#### Cucullanidae gen. sp.

**Host: *Myloplus rubripinnis* (present study).**

**Locality: Tocantins River (MA)**

#### *Cystidicoloides* sp.

**Hosts:***Psalidodon fasciatus* (=*Astyanax fasciatus*); *Hoplerythrinus unitaeniatus* (Spix & Agassiz); ***Hoplias malabaricus* (present study);** *Leporinus friderici*.

**Localities: BRAZIL:** Igarapé Fortaleza basin (a tributary of the Amazon River system) (AM); **Tocantins River, MA (present study);** Floodplain of the Upper Paraná River (PR); Upper São Francisco River (MG).

**References:**Vieira-Menezes et al. (2017), Gonçalves et al. (2016), Guidelli et al. (2011) (Cited as larvae in all these references).

#### *Goezia* sp. (larva)

**Hosts: *Boulengerella cuvieri* (present study);***Brycon orbignyanus*; *Hoplias malabaricus*; Hybrids *Colossoma macropomum* × *Piaractus mesopotamicus*; Hybrids *P. mesopotamicus* × *P. brachypomus*; *Mylossoma duriventre*; *Psalidodon fasciatus* (=*Astyanax fasciatus*); *Rhaphiodon vulpinus*; *Salminus hilarii*; *Serrasalmus marginatus* Valenciennes; *Tetragonopterus chalceus* Spix & Agassiz; *Triportheus guentheri* (Garman); *T*. *nematurus* (Kner).

**Localities: BRAZIL: Tocantins River (MA) (present study)**; Três Marias Reservoir (upper São Francisco River) (MG); Medalha lagoon (Pantanal, Corumbá), Fish farms (MS); Paraná River (Guaira and Foz do Iguaçu), Reservoir of Hydroelectric Power Station of Itaipu (Foz do Iguaçu), Upper Paraná River floodplain (PR); Marginal lagoons of the Mogi Guaçu River (Pirassununga) (SP).

**References:**Albuquerque et al. (2016), Costa-Pereira et al. (2014), Duarte et al. (2016), Fernandes et al. (2019), Guimarães et al. (2021), Jerônimo et al. (2020), Moravec et al. (1993a, 1994), Vieira-Menezes et al. (2017)

#### *Klossinemella iheringi* (Travassos, Artigas & Pereira, 1928) Costa, 1961

**Hosts:***Hoplias malabaricus*; *Hypomasticus copelandii* (=*Leporinus copelandii*); *Leporinus fasciatus* (Bloch); *Mylesinus paraschomburgkii*; ***M. paucisquamatus* (present study)**; *Myleus* sp.; *Myloplus asterias*; *Piaractus brachyopomus* (Cuvier), *Salminus hilarii*; *Schizodon nasutus*; Tetragonopterinae gen. sp.

**Localities: BRAZIL: Tocantins River (MA) (present study);** Cuiabá River, São Lourenço River (MT); Emas Experimental Station, Mogi Guaçu River, Pirassununga (SP); Trombetas River (PA).

**References:**Kohn & Fernandes (1987), Moravec & Thatcher (1997), Travassos & Kohn (1965), Travassos et al. (1928), Vicente et al. (1985).

#### *Procamallanus* (*Spirocamallanus*) *inopinatus* Travassos, Artigas & Pereira, 1928

**Hosts:***Acestrorhynchus falcatus*; *A*. *lacustris*; *Anostomoides passionis* Santos & Zuanon; *Astyanax altiparanae* Garutti & Britski; *Astyanax bimaculatus lacustris* [*sic*]; *Astyanax bimaculatus schubarti* [*sic*]*; Astyanax bimaculatus* (=*Astyanax bimaculatus bimaculatus*); *Astyanax* sp.; *Brachychalcinus orbicularis* (=*Ephyppicharax orbicularis*) (Valenciennes); *Brycon amazonicus* (Spix & Agassiz); *B*. *brevicaudatus* [*sic*]; *B*. *cephalus* (=*Brycon erythropterum*) (Günther); *B*. *hilarii* (Valenciennes); *B*. *melanopterus* (Cope); *B*. *orbygnianus* (Valenciennes); *B*. *orthotaenia* (=*Brycon lundii*); *Brycon* sp.; *Colossoma macropomum*; *Cynopotamos humeralis* [*sic*]; *Gasteropelecus sternicla* (Linnaeus); ***Hemiodus microlepis* Kner (present study)**; *Hypomasticus copelandii* (*=Leporinus copelandii*); *Hoplerythrinus unitaeniatus*; *Hoplias* aff. *malabaricus*; *Hoplias malabaricus*; *Hoplias missioneira* Rosso, Mabragaña, González-Castro, Delpiani, Avigliano, Schenone & Díaz de Astarloa; *Lebiasina multimaculata* Boulenger; *Leporinus friderici*; *L*. *maculatus* Müller & Troschel; *Leporinus* sp.; *Markiana geayi* (Pellegrin); *Megaleporinus elongatus* (=*Leporinus elongatus*) (Valenciennes); *M*. *macrocephalus* (=*Leporinus macrocephalus*) (Garavello & Britski); *M. obtusidens* (=*Leporinus obtusidens*); *M*. *piavussu* (Britski, Birindelli & Garavello); *Metynnis hypsauchen* (Müller & Troschel); *M*. *lippincottianus* (Cope); *Piaractus mesopotamicus*; *Poptella paraguayensis* (Müller & Troschel); *Psalidodon fasciatus* (=*Astyanax fasciatus*, *Astyanax fasciatus fasciatus*); *Psalidodon schubarti* (=*Astyanax schubarti*) (Britski); *Pygocentrus nattereri*; *Rhaphiodon vulpinus*; *Schizodon borellii*; ***Serrasalmus maculatus* (present study)**; *S. marginatus*; *S. spilopleura* Kner; *Triportheus angulatus* (Spix & Agassiz); *T. auritus* (Valenciennes); *T. curtus* (Garman); ***T. elongatus* (present study)**; *T. cf elongatus; T. rotundatus* (Jardine).

**Localities: ARGENTINA:** Aeroclub Pond, Riachuelo Basin, Paraná River; Riachuelo Basin, tributary of the Paraná River, Corrientes; Lagunas Perez and Totora, Riachuelo Basin; Ramada Poso Pond, Riachuelo Basin, an inflow from Paraná River; Medium Paraná River and Santa Lucia River; Pilcomayo River, Province of Salta. **BRAZIL:** Fish farms in the municipalities of Cruzeiro do Sul and Rio Branco (AC); Fish farms from lower São Francisco River (AL, SE); Manaus, floodplain lakes of the Brazilian Amazon, intensive husbandry system in a stream channel, Negro and Solimões River, Coari Lake (a tributary of the middle Solimões River) (AM); Curiaú River, fish farms, Jari River Basin, Matapi River, Vila Nova River (tributary of the Amazon River), Igarapé Fortaleza Basin (tributary of Amazon River system) (AP); Juparanã Lagoon (ES); **Tocantins River (MA) (present study);** Harmonia (Alfenas), Salobra, Swamp of Nova Ponte (municipality of Perdizes), Três Marias Reservoir (upper São Francisco River) (MG); Amambai River, Juba River, Miranda River, Negro River (a tributary of Paraguay River, Pantanal), Pantanal Mato-Grossense (Paraguay River) (MT); Curuá River (Cachimbo), Xingu River (PA); Areia (Patos) (PB); Medium Paraná River, Paraná River (Guaira and Foz do Iguaçu), reservoir of hydroelectric power station of Itaipu, upper Paraná River (PR); Guandu River (RJ); Cruzeta (RN); Machado River (Porto Velho) (RO); Batalha River, Peixe River, Pond in Aguai, Ponds of Latin American Regional Centre of Aquaculture (Pirassununga), Rio das Pedras farm (Campinas), Emas Waterfall (Mogi Guaçu River), Emas (Pirassununga), Experimental Station of Pirassununga (Mogi Guaçu River), Lakes of the Mogi Guaçu River, Paraná River (Porto Cabral) (SP). **PARAGUAY:** North of Carapegua. **PERU:** Fish farm, city of Nauta. **VENEZUELA:** Corrosal Brook near ranch Santa Marta (a tributary of the Suripá River near Palmarita); Mountain brook near San Esteban, northern Venezuela; Ranch Hato Las Mercedes, near Boca de Anaro on the Suripá River, State of Barinas.

**References:**Abdallah et al. (2012), Adriano et al. (2005), Ailán-Choke et al. (2020), Alcântara & Tavares-Dias (2015), Almeida-Berto et al. (2018), Andrade & Malta (2006), Andrade et al. (2001), Azevedo et al. (2007, 2010), Baia et al. (2018, 2019), Borges et al. (2021), Camargo et al. (2015), Carvalho et al. (2020), Corrêa et al. (2020), Dias et al. (2015), Fábio (1982), Feltran et al. (2004), Fernandes et al. (2019), Fujimoto et al. (2019), Gião et al. (2020), Gonçalves et al. (2016), Guidelli et al. (2006), Hamann (1986, 1995/1996, 1999), Kloss (1966), Kohn & Fernandes (1987), Kohn et al. (1985, 2011), Lehun et al. (2020), Martins et al. (2017), Morais et al. (2019), Moravec et al. (1993b, 1997), Moreira et al. (1994), Moreira et al. (2009, 2010), Morey & Malta (2018), Negreiros et al. (2021), Oliveira et al. (2015, 2016, 2018, 2019), Pedro et al. (2016), Pereira et al. (2018), Pereira (1935), Petter & Dlouhy (1985), Petter & Thatcher (1988), Pinto & Fernandes (1972), Pinto & Noronha (1972, 1976), Pinto et al. (1974, 1975, 1976), Ramallo et al. (2020), Ribeiro et al. (2016), Rivadeneyra et al. (2020), Santos & Tavares-Dias (2017), Santos et al. (1979), Saraiva et al. (2006a, b), Silva et al. (2011), Takemoto & Lizama (2010), Takemoto et al. (2009), Travassos & Kohn (1965), Travassos et al. (1928), Vicentin et al. (2011, 2013), Virgilio et al. (2021), Yamaguti (1961).

#### *Procamallanus* (*Spirocamallanus*) sp.

**Hosts: *Bivibranchia notata* (present study); *Brycon pesu*** Müller & Troschel **(present study); *Chalceus macrolepidotus* Cuvier (present study);** *Cynopotamus kincaidi* (Schultz); *Geophagus brasiliensis* (Quoy & Gaimard); ***Hemiodus microlepis* (present study); *H*. *unimaculatus* (present study);** *Salminus hilarii*; *Triportheus angulatus.*

**Localities: BRAZIL: Tocantins River (MA) (present study);** Três Marias Reservoir, upper São Francisco River (MG); Medium Paraná River (reservoir of the Hydroelectric Power Station of Itaipu) (PR).

**References**: Duarte et al. (2016), Kohn et al. (2011).

#### *Procamallanus* sp.

**Hosts:***Acestrorhynchus lacustris*; ***Bryconops alburnoides* Kner (present study);***Megaleporinus macrocephalus* (=*Leporinus macrocephalus*); *Psalidodon fasciatus* (=*Astyanax fasciatus*); *Schizodon altoparanae* Garavello & Britski.

**Localities: BRAZIL: Tocantins River (MA) (present study);** Aquidauana, Miranda and Paraguay Rivers (Pantanal) (MS); Upper São Francisco River (Três Marias) (MG); Upper Paraná River floodplain (PR).

**References:**Carvalho et al. (2003), Lehun et al. (2020), Santos et al. (2003), Vieira-Menezes et al. (2017).

#### *Raphidascaris* sp.

**Hosts: *Caenotropus labyrinthicus* (Kner) (present study);***Cyphocharax gilbert*; *Geophagus brasiliensis*; *Prochilodus lineatus*; *Serrasalmus* sp.

**Localities: BRAZIL:** Guandu River (RJ); Paraná River (Guaira), upper Paraná River floodplain (PR); Cruzeiro do Sul (AC); **Tocantins River (MA) (present study)**.

**References:**Abdallah et al. (2005), Moravec et al. (1993a), Virgilio et al. (2021), Lehun et al. (2020).

#### *Rhabdochona acuminata* (Molin, 1860)

**Hosts:***Acestrorhynchus britskii* Menezes; *A. lacustris*; *Astyanax asuncionensis* Géry; *A. bimaculatus*; *Barbus* sp.; *Brycon amazonicus*; *B*. *falcatus* Müller & Troschel; *B*. *melanopterus*; *B*. *orbignyanus*; ***B*. *pesu* (present study);** *Bryconamericus iheringi* (Boulenger); cichlid sp. [*sic*]; *Hoplias* aff. *malabaricus*; *Leporellus vittatus*; *Leporinus friderici*; *L*. *pearsoni* Fowler; *Megaleporinus macrocephalus* (=*Leporinus macrocephalus*); *Myloplus rubripinnis*; *Psalidodon fasciatus* (=*Astyanax fasciatus*); *P*. *schubarti* (=*Astyanax schubarti*) (Britski); *Salminus hilarii*; Tetragonopterinae gen. sp.; *Tetragonopterus argenteus* Cuvier; *Tetragonopterus* sp.; *Triportheus angulatus*; *T*. *auritus*; *T*. *nematurus*; ***T*. *trifurcatus*** (**present study**).

**Localities: ARGENTINA:** El Tunal Reservoir, Salta; Medina River, Province of Tucuman. **BRAZIL:** fish farms in the municipalities of Cruzeiro do Sul and Rio Branco (AC); Solimões River (AM); Jari River (AP); **Tocantins River (MA) (present study);** Três Marias Reservoir (upper São Francisco river) (MG); Mato Grosso (MT); Upper Paraná River (PR); Lajes Reservoir (RJ); Lake Guaiba (RS); Experimental Station (Mogi Guaçu River, Pirassununga), Mogi Guaçu River, Paranapanema River, Peixe River, Taquari River, Tietê River, Veados River, Lakes of Mogi Guaçu River (SP). **ECUADOR:** Hacienda Primavera, Napo River; San Pablo Kantesya, Aguarico River, Province of Napo.

**References:**Abdallah et al. (2012), Borges et al. (2021), Cancino & Ramallo (2008) *apud*Ramallo & Cancino (2021), Corrêa et al. (2020), Costa et al. (2011), Duarte et al. (2016), Fernandes et al. (2019), Gallas et al. (2019), Kloss (1966), Kohn & Fernandes (1987), Martins et al. (2017), Molin (1860), Negreiros et al. (2021), Paraguassú & Luque (2007), Petter (1987), Ramallo (2005), Ribeiro et al. (2016), Travassos & Kohn (1965), Travassos et al. (1928), Vicente et al. (1985), Virgilio et al. (2021), Yamada et al. (2017).

#### *Rhabdochona* sp.

**Hosts:***Brycon orthotaenia*; *Gymnocorymbus ternetzi* (Boulenger); *Leporinus octofasciatus* Steindachner; *Psalidodon fasciatus* (=*Astyanax fasciatus*); *Salminus brasiliensis*; *Tetragonopterus argenteus*; ***Triportheus elongatus* (present study).**

**Localities: BRAZIL: Tocantins River (MA) (present study);** Três Marias Reservoir, upper São Francisco River (MG); Fish farm (SC); Emas (Pirassununga), Mogi Guaçu River, Pirassununga (SP).

**References**: Kohn & Fernandes (1987), Luque et al. (2011), Pinto et al. (2010), Mesquita et al. (2012), Santos et al. (2017), Vieira-Menezes et al. (2017)

#### *Rondonia rondoni* Travassos, 1920

**Hosts:** Hybrid *P. mesopotamicus* x *P. brachypomus*; ***Leporinus fasciatus* (present study); *Leporinus* sp. (present study);** *Mylesinus paraschomburgkii*; ***M*. *paucisquamatus* (present study);** *Myletes* sp.; *Myleus micans*; *Myleus* sp.; *Myloplus asterias* (=*Myleus asterias*); *M*. *torquatus* (=*Myleus torquatus*); *Piaractus brachypomus*; *P*. *mesopotamicus* (= *Colossoma mitrei*); *Rhaphiodon vulpinus*; *Salminus* sp.; *Tometes camunani* Andrade, Giarrizzo & Jégu.

**Localities: ARGENTINA:** Medium Paraná River, Corrientes. **BRAZIL:** Uatumã, Pitinga, Capucapu River (AM); Araguari River (AP); **Tocantins River (MA) (present study);** Aquidauana, Miranda and Paraguai Rivers (Pantanal), Porto Esperança (Paraguai River) (MS); São Francisco River (Três Marias) (MG); Cuiabá River (São João), Cuiabá and Paraguai Rivers (Pantanal), Salobra (MT); Emas Experimental Station (Pirassununga), fish farm of CEPTA (Pirassununga), fish farms from northwest of São Paulo State, ponds of CERLA (Pirassununga), Emas waterfall (Pirassununga), Mogi-Guaçu River (Emas), Mogi-Guaçu River (Reservoir of Aquaculture Center at Unesp, Jaboticabal), Porto Cabral (Paraná River) (SP); Jari River, Trombetas River Basin (PA); Medium Paraná River (Hydroelectric Power Station of Itaipu) (PR). **PERU:** Fish farm located in the city of Nauta.

**References:**Andrade et al. (2013), Brasil-Sato & Santos (2003), Campos et al. (2009), Cuadros et al. (2021), Franceschini et al. (2013a), Hamann (1982a), Kohn & Fernandes (1987), Kohn et al. (1985, 2011), Luque et al. (2011), Martins & Urbinati (1993), Masi Pallares et al. (1973), Parra et al. (1997), Rêgo & Vicente (1988), Santos et al. (2003), Thatcher & Jégu (1996), Travassos (1920, 1923, 1940, 1945), Travassos & Freitas (1943), Travassos & Kohn (1965), Travassos et al. (1928), Travassos et al. (1939).
